# Rate-Independent Constructs for Chemical Computation

**DOI:** 10.1371/journal.pone.0021414

**Published:** 2011-06-30

**Authors:** Phillip Senum, Marc Riedel

**Affiliations:** Department of Electrical and Computer Engineering, University of Minnesota, Minneapolis, Minnesota, United States of America; Center for Genomic Regulation, Spain

## Abstract

This paper presents a collection of computational modules implemented with chemical reactions: an inverter, an incrementer, a decrementer, a copier, a comparator, a multiplier, an exponentiator, a raise-to-a-power operation, and a logarithm in base two. Unlike previous schemes for chemical computation, this method produces designs that are dependent only on coarse rate categories for the reactions (“fast” vs. “slow”). Given such categories, the computation is exact and independent of the specific reaction rates. The designs are validated through stochastic simulations of the chemical kinetics.

## Introduction

The theory of reaction kinetics underpins our understanding of biological and chemical systems [1]. It is a simple and elegant formalism: chemical reactions define *rules* according to which reactants form products; each rule fires at a *rate* that is proportional to the quantities of the corresponding reactants that are present. On the computational front, there has been a wealth of research into efficient methods for simulating chemical reactions, ranging from ordinary differential equations (ODEs) [2] to stochastic simulation [3]. On the mathematical front, entirely new branches of theory have been developed to characterize the dynamics of chemical reaction networks [4].

Most of this work is from the vantage point of *analysis*: a set of chemical reaction exists, designed by nature and perhaps modified by human engineers; the objective is to understand and characterize its behavior. Comparatively little work has been done at a conceptual level in tackling the inverse problem of *synthesis*: how can one design a set of chemical reactions that implement specific behavior?

Of course, chemical engineers, genetic engineers and other practitioners strive to create novel functionality all the time. Generally, they begin with existing processes and pathways and modify these experimentally to achieve the desired new functionality [5], [6]. In a sense, much of the theoretical work on the dynamics of chemical reactions also addresses the synthesis problem by delineating the range of behaviors that are possible. For instance, theoretical work has shown that fascinating oscillatory and chaotic behaviors can occur in chemical reaction networks [7], [8].

Perhaps the most profound theoretical observation is that chemical reaction networks are, in fact, *computational processes*: regardless of the complexity of the dynamics or the subtlety of the timing, such networks transform *input* quantities of chemical species into *output* quantities through simple primitive operations. The question of the computational power of chemical reactions has been considered by several authors. Magnasco demonstrated that chemical reactions can compute anything that digital circuits can compute [9]. Soloveichik *et al.* demonstrated that chemical reactions are *Turing Universal*, meaning that they can compute anything that a computer algorithm can compute [10].

Such prior work considered the computational power of chemical reactions from a *deductive* point of view. This paper tackles the problem from an *inductive* point of view. We present a constructive method for designing specific computational modules: an inverter, an incrementer, a decrementer, a copier, a comparator, a multiplier, an exponentiator, a raise-to-a-power operation, and a logarithm in base two. This work builds upon our prior work that described constructs such as “for” and “while” loops [11] and signal processing operations such as filtering [12].

In contrast to previous work, our method produces designs that are dependent only on coarse rate categories for the reactions (e.g., “fast” and “slow”). It does not matter how fast any “fast” reaction is relative to another, or how slow any “slow” reaction is relative to another – only that “fast” reactions are fast relative to “slow” reactions. Specifically, suppose that we design a module that requires 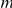 slow reactions and 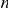 fast reactions. Any choice of 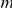 reactions with kinetic rate constants 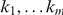 and 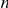 reactions with kinetic rate constants 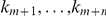, where 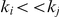, for all 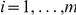, for all 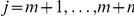, will work.

The result of the computation is *rate-independent* in the sense that the *formula* of what is computed, say a logarithm, does not include any of the kinetic rate constants. We do not mean to imply that the rates do not matter. If the separation between “slow” and “fast” is not sufficiently large, then errors will occur. However, for a sufficiently large separation, the errors are small.

Indeed, the error that occurs as a function of the separation between “fast” and “slow” is our main criterion of goodness for our design. As Tables 1, 2, 3, 4, 5, 6 illustrate, our constructs perform remarkably well, computing with small errors for rate separations of 100 or 1,000 and vanishingly small errors for rate separations of 10,000. We validate our all of our designs through stochastic simulations of the chemical kinetics [13] using an open-source tool called Cain [14]. (Details about Cain can be found in the section “Analysis”.)

**Table 1:** Logical operations via chemical reactions.

Operation	Creation	Destruction	Operation	Creation	Destruction
a = = b	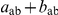	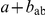	a> = b	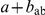	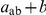
		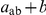		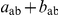	
a>b	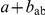	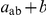	a< = b	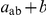	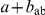
		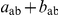		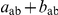	
a<b	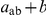	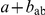	a! = b	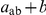	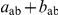
		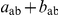		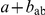	

**Table 2:** Statistical simulation results for “Multiplier” construct.

Trial	Rate Separation	Trajectories	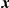	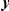	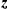	Expected 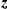	Error
1	100	100	100	50	4954.35	5000	0.91%
2	100	100	50	100	4893.18	5000	2.14%
3	1000	100	100	50	4991.56	5000	0.17%
4	1000	100	50	100	4995.78	5000	0.08%
5	10000	100	100	50	4998.69	5000	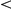 0.01%
6	10000	100	50	100	4999.14	5000	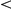 0.01%
7	10000	100	10	20	200.04	200	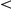 0.01%
8	10000	100	20	10	200.03	200	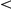 0.01%

**Table 3:** Statistical simulation results for “Copier” construct.

Trial	Rate Separation	Trajectories	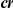	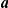	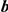	Expected 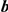	Error
1	100	500	5	100	102.45	100	2.45%
2	100	500	50	100	104.826	100	4.826%
3	1000	500	5	100	100.312	100	0.312%
4	1000	500	50	100	100.516	100	0.516%
5	10000	500	5	100	100.022	100	0.022%
6	10000	500	50	100	100.034	100	0.034%
7	10000	500	5	5000	4938.39	5000	1.232%
8	10000	500	50	5000	4967.26	5000	0.655%
9	10000	500	200	5000	4796.38	5000	4.072%
10	10000	500	50	2	2	2	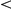 0.01%

**Table 4:** Statistical Simulation Results from “Raise to a Power” Construct.

Trial	Rate Separation	Trajectories	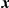	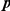	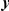	Expected 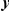	Error
1	10000	100	3	9	19734.3	19683	0.26%
2	10000	100	4	8	64884.7	65536	0.99%
3	10000	100	5	4	626.87	625	0.30%
4	10000	100	6	7	279864	279936	0.03%
5	10000	100	9	6	531412	531441	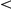 0.01%
6	10000	100	10	3	999.43	1000	0.06%

**Table 5:** Statistical Simulation Results from “Exponentiation” Construct.

Trial	Rate Separation	Trajectories	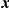	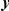	Expected 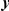	Error
1	10000	100	2	4	4	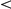 0.01%
2	10000	100	3	8	8	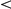 0.01%
3	10000	100	6	64.32	64	0.50%
4	10000	100	9	514.3	512	0.45%
5	10000	100	11	2051.48	2048	0.17%
6	10000	100	19	523461	524228	0.15%

**Table 6:** Statistical Simulation Results from “Logarithm” Construct.

Trial	Rate Separation	Trajectories	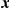	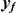	Expected 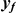	Error
1	10000	100	2	1	1	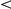 0.01%
2	10000	100	10	3	3	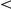 0.01%
3	10000	100	62	5	5	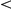 0.01%
4	10000	100	83	6	6	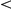 0.01%
5	10000	100	163	7	7	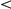 0.01%
6	10000	100	286	7.99	8	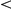 0.01%
7	10000	100	1165	10	10	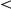 0.01%

### Chemical Model

We adopt the model of discrete, stochastic chemical kinetics [3], [15]. Molecular quantities are whole numbers (i.e., non-negative integers). Reactions fire and alter these quantities by integer amounts. The reaction rates are proportional to (1) the quantities of the reacting molecular types; and (2) kinetic rate constants. As discussed above, all of our designs are formulated in terms of two coarse kinetic rate constant categories (“fast” and “slow”).

Consider the reaction
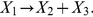
(1)When this reaction fires, one molecule of 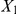 is consumed, one of 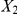 is produced, and one of 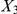 is produced. (Accordingly, 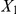 is called a *reactant* and 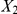 and 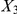 the *products*.) Consider what this reaction accomplishes from a computational standpoint. Suppose that it fires until all molecules of 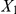 have been consumed. This results in quantities of 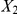 and 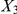 equal to the original quantity of 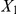, and a new quantity of 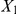 equal to zero:
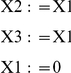



Consider the reaction
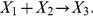
(2)Suppose that it fires until either all molecules of 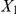 or all molecules of 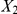 have been consumed. This results in a quantity of 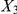 equal to the lesser of the two original quantities:
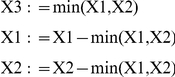



We will present constructs different arithmetical and logical operations in this vein. Each sets the final quantity of some molecular type as a function of the initial quantities of other types.

Most of our designs consist of either unimolecular or bimolecular reactions, i.e., reactions with one or two reactants, respectively. A small subset of the reactions are trimolecular. Mapping these to chemical substrates might not be feasible, since the kinetics of reactions with more than two reactants are complex and often physically unrealistic. For all trimolecular reactions, we suggest the follow generic scheme for converting them into bimolecular reactions. (This idea is found in [16] in the context of DNA strand displacement reactions.) We convert any trimolecular reaction
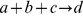
(3)into a pair of reactions
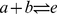
(4)

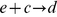
(5)where 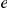 is an new intermediary type. Note that Reaction 4 is a reversible reaction. We assume that this reaction is fast relative to all others. Accordingly, if there are non-zero quantities of 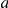 and 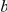 but zero of 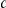, the system will “back-off”, converting 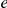 back into 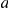 and 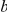. Other reactions in the system that use 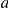 and 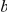 can continue to fire.

Apart from reactions resulting from such trimolecular conversions, we do not use reversible reactions in any of our constructs. Of course, all chemical reactions are reversible. Implicitly, we assume that all reverse rates are much slower that the forward reactions (except for those corresponding to Reaction 4).

## Results

### Computational Constructs

In this section, we present a collection of constituent constructs for rate-independent computation: an inverter, an incrementer/decrementer, a copier, and a comparator. In the next section, we use some of these constructs to implement a multiplier, a logarithm operation, an exponentiator, and a raise-to-the-power operation. A reference of all reactions needed for these constructs can be found in Supporting Information S1.

### An Inverter

We implement an operation that is analogous to that performed by an inverter (i.e., a NOT gate) in a digital system: given a non-zero quantity (corresponding to logical “1”) we produce a zero quantity (corresponding to logical “0”). Conversely, given a zero quantity, we produce a non-zero quantity. We accomplish this with a pair of chemical types: the given type, for example, 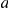, and a corresponding “**absence indicator**” type, which will be referred to as 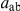. The reactions generating the absence indicator are shown in reactions 6–8.
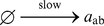
(6)

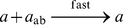
(7)

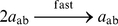
(8)Note that when the empty set symbol, 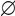, is used as a reactant, it indicates that the reactants are a large or replenishable unreactive source; when it is used as a product, it indicates that the products of the reaction are waste.

The first reaction continuously generates molecules of 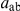, so in the absence of molecules of 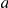 we will have a non-zero quantity of 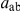 in the system. If there are molecules of 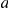 present, then second reaction quickly consumes any molecules of 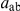 that are generated, so the quantity of 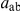 will be close to zero. The third reaction ensures that the quantity 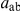 remains small.

We use this simple construct in many of our computational modules [12], [17]. It is also a fundamental part of all of the constructs introduced in this paper. In general, it can be used to synchronize steps. Suppose that we want to perform an operation similar to the one in reactions 9–10.
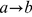
(9)

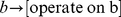
(10)Here the second step is an operation that depends on the quantity of 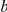. We do not want to start consuming molecules of 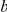 until the full quantity of it is generated from 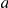. We can accomplish this with an absence indicator 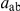:
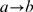
(11)

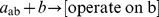
(12)


It is important to note that absence indicators generated by reactions 6–8 can only be used with “slow” reactions. If they were used by a “fast” reaction, it is possible that a false positive could be detected because a “fast” will compete with reaction 7. In situations where absence indicators need to be consumed by “fast” reactions, we can use an alternate two-step process to produce them.
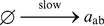
(13)

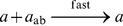
(14)

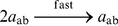
(15)

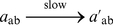
(16)

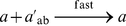
(17)

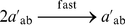
(18)In this case, a secondary absence indicator, 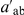 is produced from 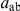 through a “slow” reaction. This allows “fast” reactions to use 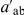 safely because it is buffered through reaction 16.

### Increment and Decrement Operations

We describe constructs to implement incrementation and decrementation. These operations form the basis of more complex arithmetical operations, such as multiplication. The inputs consist of two molecular types: 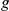, the “start signal;” and 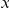, the quantity to be incremented or decremented. We assume that some external source injects molecules of 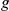. Any quantity can be injected; regardless, the quantity of 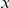 is incremented or decremented by exactly one, consuming all the molecules 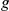 in the process. The operations proceed as follows:

1. The system waits for the start signal 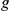 to be some non-zero quantity.
2. It transfers the quantity of 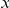 to a temporary type 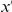.
3. It sets 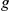 to zero.
4. It transfers all but one molecule of 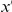 back to 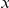.
5. For a decrement, it removes the last molecule 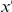.
6. For an increment, it removes the last molecule of 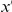 and adds to two molecules to 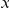.

The following reactions implement this scheme. Given molecules of 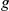, a reaction transfers molecules of 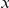 to molecules of 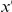:
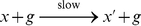
(19)The following reaction sets the quantity of 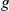 to zero. Using the absence indicator mechanism described in the preceding section, it does so only once all molecules of 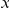 have been transfered to 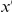:
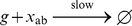
(20)Reactions of the form of 6–8 are needed to generate 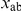; we omit them here. The following reaction transfers all but one molecule of 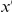 back to 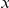. It does so by repeatedly selecting pairs of 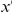 and turning one molecule of 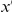 into 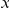. In essence, this is a repeated integer division by two. Again, using the absence indicator mechanism, it proceeds only once all molecules of 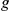 have been removed:
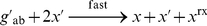
(21)In reaction 21, we do not directly use an absence indicator for 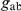, but instead, we use a secondary absence indicator 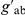, generated in the method outlined in reactions 13–18.

Reaction 21 also produces molecules of a supplementary type 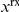. Note that this reaction is in the “fast” category. The new type 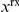 is consumed by the reaction:
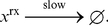
(22)Note that this reaction is in the “slow” category. We introduce 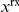 because we cannot directly use an absence indicator for 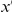 to detect when reaction 21 has completed because 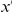 is never completely consumed. Instead, we use 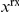 to indicate that we are currently transferring molecules of 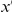 back to 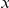; it is consumed when the step completes. Again, reactions of the same form as 6–8 are needed to generate 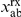; we omit them here.

Finally, we include the following reaction to perform a decrement:
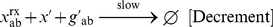
(23)Or we include the following reaction to perform an increment:
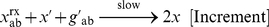
(24)With a slight modification of reaction 21, we can also implement division by 2 with this module:
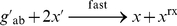
(25)


### A Copier

In digital computation, one of the most basic operations is copying a quantity from one register into another. The programming construct is “set the value of 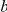 to be the value of 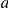
”:
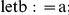
To implement an equivalent operation with chemical reactions, we could use a reaction that simply transfers the quantity of 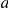 to 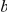:
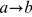
(26)However, this is not ideal because this reaction consumes all the molecules of 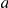, setting its quantity to zero. We would like a chemical construct that copies the quantity without altering it. The following reaction does not work either:
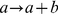
(27)It just creates more and more molecules of 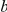 in the presence of 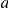. A more sophisticated construct is needed.

In our construct, we have a “start signal” type 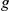. When an external source injects molecules of 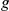, the copy operation proceeds. (In the same way as our increment and decrement operations, the quantity of 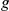 that is injected is irrelevant.) It produces an output quantity of 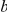 equal to the input quantity of 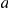; it leaves the quantity of 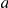 unchanged.

The reactions for the copier construct are as follows. Firstly, in the presence of 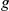, a reaction transfers the quantity of 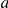 to 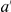:
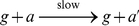
(28)Secondly, after all molecules of 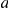 have been transferred to 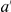, the system removes all the molecules of 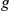:
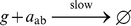
(29)Here, again, we are using the concept of an absence indicator. Removing 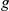 ensures that 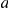 is copied exactly once.

After 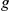 has been removed, a reaction transfers the quantity of 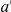 back to 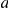 and also creates this same quantity of 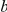:
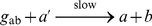
(30)Alternatively, we can use a slight modification of this reaction to double the quantity of 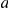:
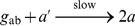
(31)


We also generate absence indicators 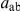 and 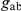 by the same method as reactions 6–8.

We note that, while this construct leaves the quantity of 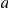 unchanged after it has finished executing, it temporarily consumes molecules 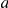, transferring the quantity of these to 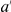 before transferring it back. Accordingly, no other constructs should use 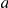 in the interim.

### A Comparator

Using our copier construct, we can create a construct that compares the quantities of two input types and produces an output type if one is greater than the other. For example, let us assume that we want to compare the quantities of two types 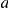 and 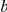:
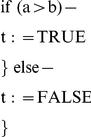



If the quantity of 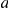 is greater than the quantity of 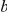, the system should produce molecules of an output type 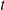; otherwise, it should not produce any molecules of 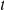.

First, we create temporary copies, 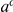 and 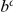, of the types that we wish to compare, 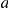 and 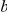, using the copier construct described in the previous section. (We omit these reactions; they are two verbatim copies of the copier construct, one with 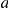 as an input and 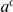 as an output, the other with 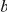 as an input and 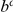 as an output.) We split the start signal so that the two copiers are not competing for it:
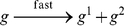
(32)


Now we compare 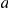 and 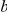 via their respective copies 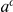 and 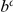. To start, we first consume pairs of 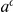 and 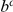:
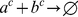
(33)We assume that this reaction fires to completion. The result is that there are only molecules of 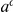 left, or only molecules of 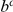 left, or no molecules of 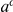 nor 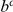 left. Molecules of the type that originally had a larger quantity have persisted. If the quantities were equal, then both types were annihilated. We use absence indicators 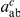 and 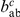 to determine which type was annihilated, produced by the method shown in reactions 6–8. If 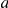 was originally greater than 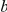, there will now be a presence of 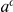 and an absence of 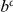. We produce molecules of type 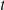 if this condition is met. We preserve the quantities of 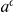 and 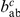. We can also limit the quantity of 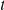 produced by introducing a fuel type:
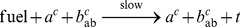
(34)For robustness, we also add reactions to destroy 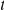 in the case that the asserted condition is not true:
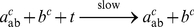
(35)

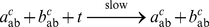
(36)We can readily generalize the construct to all types of logical comparisons. Table 1 lists these operations and their corresponding reactions.

### Complex Arithmetic

Based upon the modules described in the previous section, we provide examples of how to implement more complex arithmetic: multiplication, logarithms in base two, exponentiation, and raising to a power. In order to elucidate the designs, we specify the sequence of operations for each of these module in pseudo-code. The pseudo-code operations consist of:

- Assignment, addition, and subtraction operations. The operands may be constants or variables.
- Decision-making constructs: while and if statements. The logical test for each of these constructs can be any one of the six conditions listed in Table 1. In some cases, the if and while statements will be nested.

#### A Multiplier

Building upon the constructs in the last section, we show a construct that multiplies the quantities of two input types. Multiplication can be implemented via iterative addition. Consider the following lines of pseudo-code:
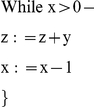
The result is that 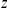 is equal to 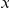 times 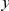. We implement multiplication chemically using the constructs described in the previous sections: the line z = z+y is implemented with a copy operation; the line x = x−1 is implemented using a decrement operation. A third set of reactions handle the looping behavior of the while statement. The reactions presented here are also listed in Supporting Information S2.

Firstly, we have reactions that copy the quantity of 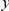 to 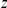. We use “start signal” types 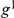 and 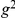 to synchronize iterations; it is supplied from the controlling reaction 48 below.
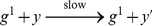
(37)

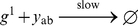
(38)

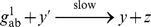
(39)Secondly, we have reactions that decrement the value of 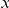. We use 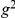 as the signal to begin the decrement.
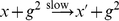
(40)

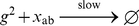
(41)

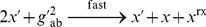
(42)

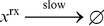
(43)

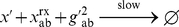
(44)Thirdly, we have a controlling set of reactions to implement the while statement. This set generates 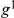 and 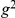 to begin the next iteration, preserving the quantity of 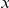:
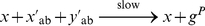
(45)

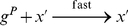
(46)

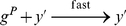
(47)

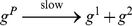
(48)This set initiates the next iteration of the loop if such an iteration is not already in progress and if there are still molecules of 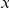 in the system. The types 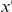 and 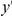 are present when we are decrementing 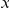 or copying 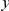, respectively; thus, they can be used to decide whether we are currently inside the loop or not. Finally, we generate the four absence indicators according to the template in reactions 6–8.

#### Raise to a Power

**Figure 1:**
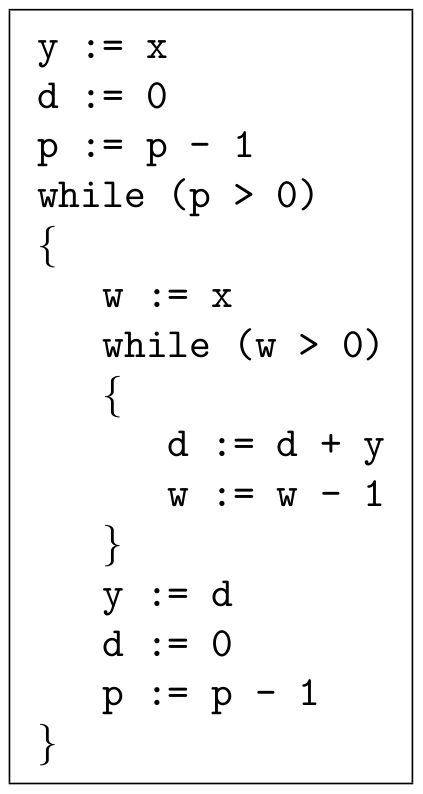
Pseudo-code to implement raise-to-a-power operation.

As a second complex example, we show how to implement the operation 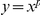. This can be done using iterative multiplication; as we demonstrated in the previous section, multiplication can be implemented via iterative addition. The pseudo-code for the raising-to-a-power operation is shown in Figure 1. It consists of assignment, addition, subtraction, and iterative constructs. Note that the assignment operations can be performed with our “copier” module; the addition and subtraction operations can be performed with “increment” and “decrement” modules. A pair of nested while constructs, similar to that used for multiplication, perform the requisite iterative computation. The complete set of reactions to implement this operation is given in Supporting Information S3.

#### Exponentiation

**Figure 2:**
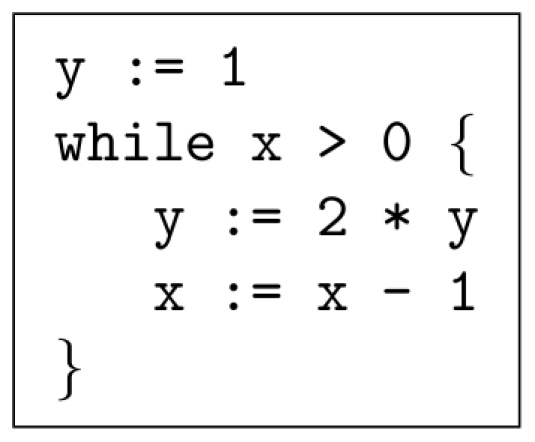
Pseudo-code to implement the exponentiation operation.

To implement the operation 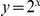, we can use a sequence of operations similar to those that we used for multiplication. The pseudo-code is shown in Figure 2. The reactions that implement this pseudo-code are given in Supporting Information S4.

#### Logarithm

**Figure 3:**
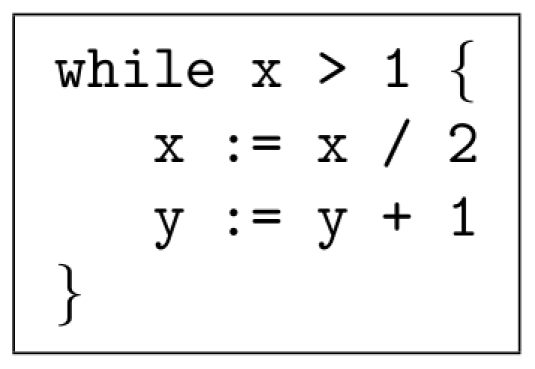
Pseudo-code to implement the logarithm operation.

We demonstrate the computation of a base-2 logarithm. The pseudo-code is shown in Figure 3. A logarithm is the inverse operation of exponentiation; it makes sense, therefore, that the pseudo-code for 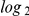 is more or less the reverse of that for exponentiation. The reactions that implement this operation are given in Supporting Information S5.

### Simulation Results

We validated our constructs using stochastic simulation. Specifically, we performed a time homogeneous simulation using Gillespie's “Direct Method” [3] with the software package “Cain” from Caltech [14]. (Details about Cain can bew found in the section “Analysis”.) In each case, the simulation was run until the quantities of all types except the absence indicators converged to a steady state. We used a rate constant of 1 for the “slow” reactions. We tried rate constants between two to four orders of magnitude higher for the “fast” reactions. (We refer to the ratio of “fast” to “slow” as the *rate separation*.) For each of the graphs below, the initial quantity of each type is zero, with the exception of the types specified.

#### Multiplier

**Figure 4:**
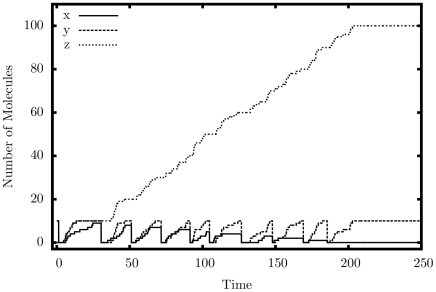
Simulation output of the multiplication construct. 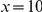
, 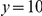.

Figure 4 shows the output of a single simulated trajectory for our multiplier. We observe exactly the behavior that we are looking for: the quantity of 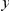 cycles exactly 10 times as it exchanges with 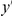 and is copied to 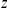; the quantity of 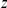 grows steadily up to 100; the quantity of 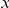 decreases once each cycle down to 0. Table 1 presents detailed simulation results, this time tested for accuracy. Errors generally occur if the system executes too many or too few iterations. As can be seen, the larger the quantity of 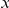, the more accurate the result, in relative terms. As expected, the larger the rate separation, the fewer errors we get.

#### Copier

**Figure 5:**
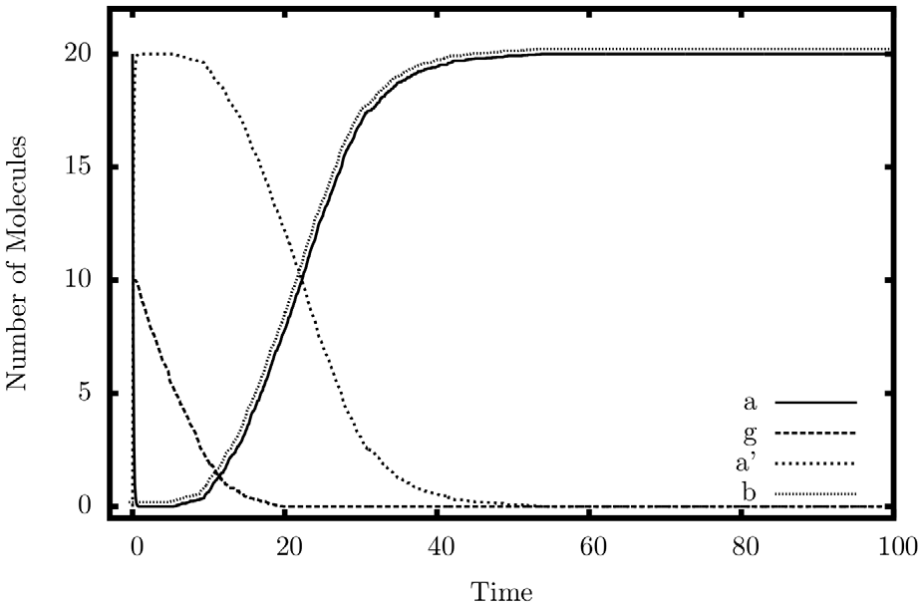
Simulation output of the copier construct. 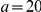
, 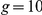.

Figure 5 shows an average simulated trajectory for our copier. Again, we observe exactly the behavior we expect: the quantity of 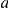 drops to 0 almost immediately as it turns into 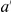; this is followed by the removal of 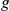 from the system. When the quantity of 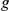 drops to nearly zero, both 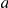 and 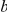 rise steadily back to the original quantity of 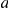. Table 2 shows additional simulation results from our copier, this time tested for accuracy. The copier construct appears to be quite robust to errors; however, large rate separations do not help as much as they do for the multiplier. The system seems to prefer a larger injection quantity of 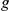, but whether it is larger or smaller than the initial quantity of 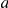 is irrelevant.

#### Decrementer

**Figure 6:**
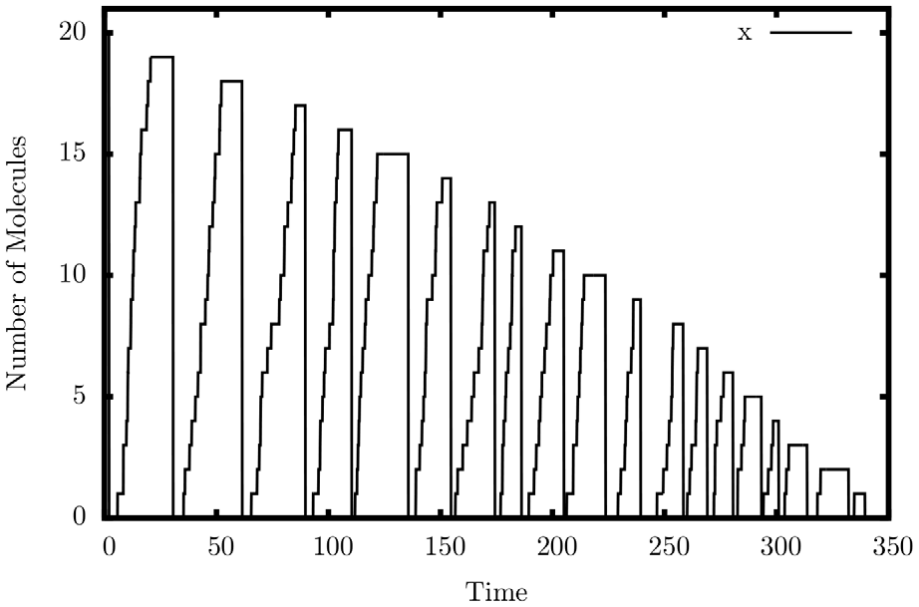
Simulation output of the decrement construct. 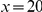
.

Figure 6 shows the output of a single simulated trajectory of our decrementer. An automatic restart mechanism, similar to reactions 45–48, was used to produce a continuous series of decrements. Exactly twenty peaks can be seen in the graph, including the initial peak on the far-left margin of the graph. This is exactly the behavior we are looking for – a decrement by exactly one each cycle.

#### Comparator

**Figure 7:**
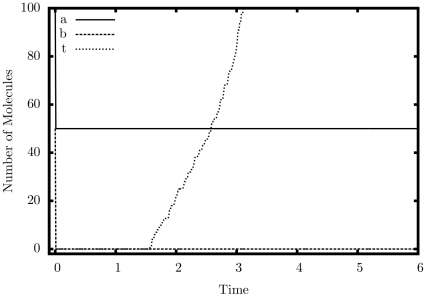
Simulation output of the comparator (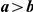) construct. 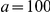
, 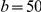.

**Figure 8:**
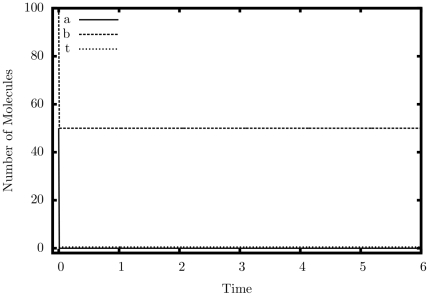
Simulation output of the comparator (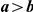) construct. 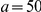
, 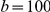.

Figures 7 and 8 display simulation results from our comparator. In Graph 4, 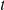 is asserted as we would expect; in Graph 5, 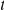 is not asserted, also as we would expect.

#### Raise to a Power

**Figure 9:**
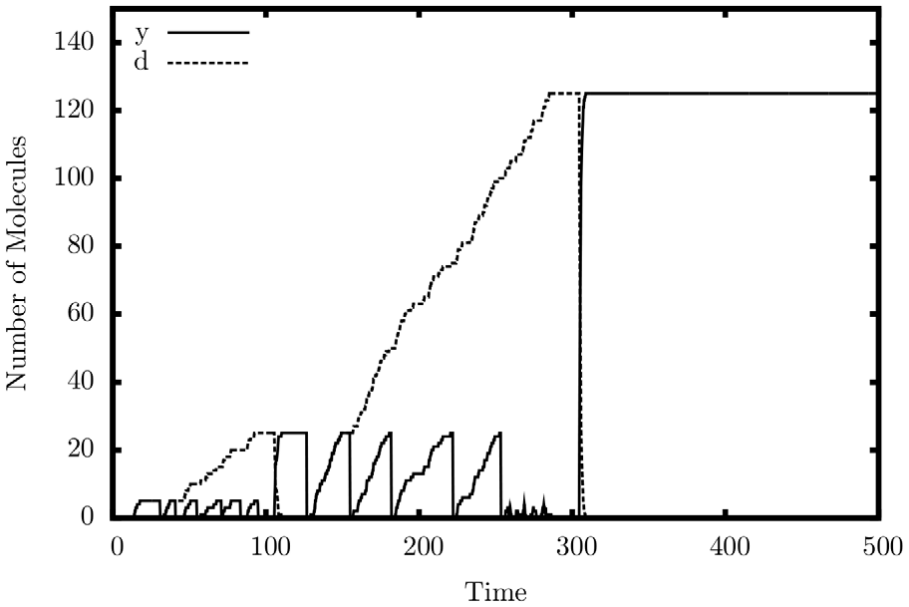
Simulation output of the raise-to-power construct. 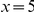
, 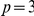, 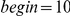.

Figure 9 shows a simulated trajectory of our raise-to-a-power construct. As can be observed, after 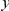 is loaded with the initial quantity of 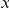, it is multiplied by 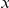 twice. Each time its value is stored in the temporary type 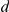 before being transferred back. Table 4 shows simulation results for our raise-to-a-power construct for various values of 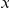 and 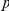. In each case, the initial quantity of 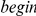 was set to 10, simulating an injection of that type.

#### Exponentiation

**Figure 10:**
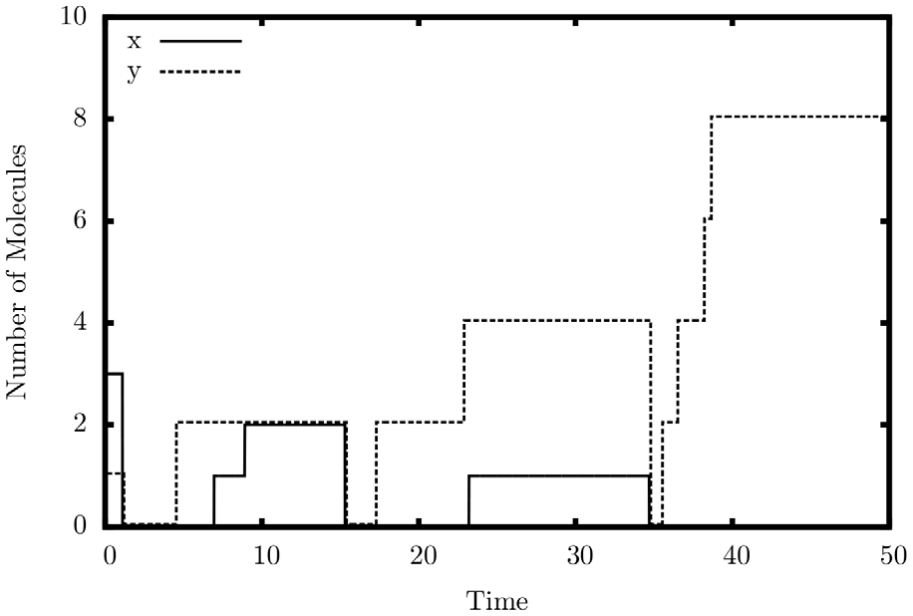
Simulation output of the exponentiation construct. 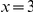
, 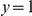.

Figure 10 shows a simulated trajectory of our exponentiation construct. We can observe that for every decrementation of 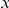, 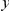 doubles in value, which is the behavior that we are aiming for. Table 5 shows more simulation results. The error for this construct is small but appears to grow as 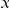 grows. This is not surprising, given we are performing exponentiation: small errors will be compounded.

#### Logarithm

**Figure 11:**
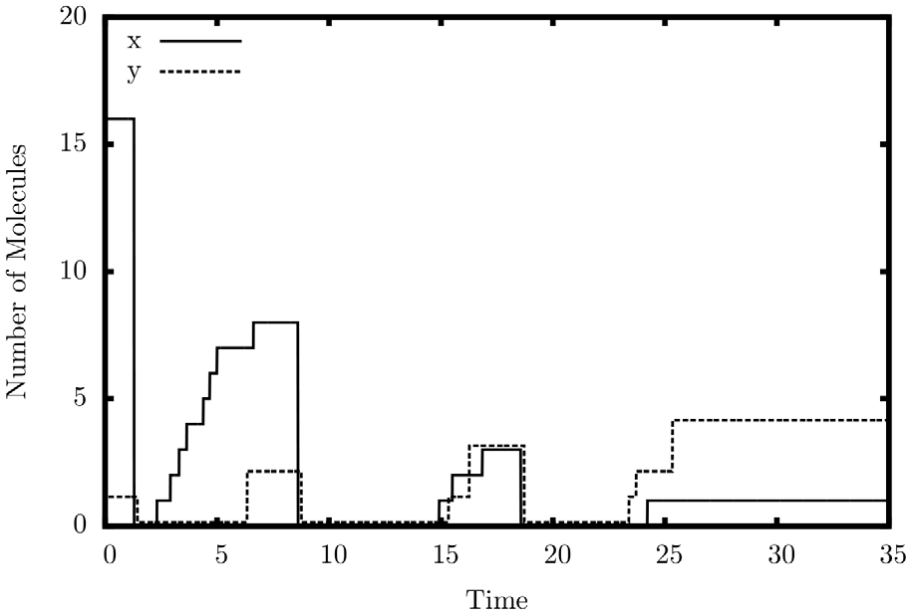
Simulation output of the logarithm construct. 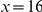
, 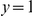.

Figure 11 shows a simulated trajectory for our base-2 logarithm construct. Again, we observe the behavior that we are expecting; every time we divide 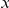 by two, 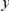 increases by one. Table 6 shows more detailed simulation results.

## Discussion

This paper presented a collection of specific computational constructs. More complex operations – multiplication, exponentiation, raising to a power, and logarithms – were built a collection of robust, primitive operations – absence indicators, incrementing and decrementing, copying, and comparing. The process by which we assembled these primitive operations could be readily generalized. Indeed, we are developing a chemical compiler that will translate any sequence of operations specified by pseudo-code into chemical reactions. The compiler will accept general pseudo-code written in the vein of that shown in Figures 1, 2, 3. It will allow for assignments, arithmetic operations, “if” statements, and arbitrarily nested “while” loops.

The novelty and value of the constructs that we have demonstrated is that they are all rate independent. Here “rate independent” refers to the fact that, within a broad range of values for the kinetic constants, the computation does not depend on the specific values of the constants. Of course, outside of this range, the accuracy of the computation degrades. For rates within the target range, our results are remarkably accurate: in nearly all cases the errors are less than 1%. In many cases, the errors are much less than 1%. The actual value of the target range will depend on the chemical substrate used; in simulation, it was found that a ratio of 10,000∶1 of “fast” vs. “slow” produced nearly perfect results.

Our contribution is to tackle the problem of synthesizing computation at a conceptual level, working not with actual molecular types but rather with abstract types. One might question whether actual chemical reactions matching our templates can be found. Certainly, engineering complex new reaction mechanisms in any experimental domain is formidable task; for *in vivo* systems, there are likely to be many experimental constraints on the choice of reactions [18]. However, we point to recent work on *in vitro* computation as a potential application domain for our ideas.

Through a mechanism called DNA strand-displacement, a group at Caltech has shown that DNA reactions can emulate the chemical kinetics of nearly any chemical reaction network. They also provide a compiler that translates abstract chemical reactions of the sort that we design into specific DNA reactions [16]. Recent work has demonstrated both the scale of computation that is possible with DNA-based computing [19], as well as exciting applications [20]. While conceptual, our work suggest a *de novo* approach to the design of biological functions. Potentially this approach is more general in its applicability than methods based on appropriating and reusing existing biological modules.

## Analysis

All the models described in this paper are contained in an XML file. This file is available at:
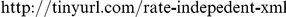



The file is designed for use with Cain, a biochemical simulator from Caltech [14]. It contains initial quantities for all types. All non-zero quantities can be modified as the user desires to simulate different input values. Within Cain, we suggest *Gillespie's Direct Method* for all simulations.

## Supporting Information

Supporting Information S1 Appendix: Module Reference.
(PDF)

Supporting Information S2 Appendix: Multiplication Reactions.
(PDF)

Supporting Information S3 Appendix: Raise-to-a-Power Reactions.
(PDF)

Supporting Information S4 Appendix: Exponentiation Reactions.
(PDF)

Supporting Information S5 Appendix: Logarithm Reactions.
(PDF)
