# Supplementary material for: Rate-Independent Constructs for Chemical Computation
Source: PLoS One. 2011 Jun 30;6(6):e21414. doi: 10.1371/journal.pone.0021414 (PMC3127954; doi:10.1371/journal.pone.0021414)
Supplement: Supporting Information S1 — Appendix: Module Reference. (PDF) [file pone.0021414.s001.pdf]

Rate-Independent Constructs for Chemical Computation

Phillip Senum, Marc Riedel
Department of Electrical and Computer Engineering, University of Minnesota, Minneapolis, Minnesota, United States
E-mail: { senu0004, mriedel }@umn.edu

Appendix: Module Reference

Absence Indicator

Generates a_{ab} in the absence of a, and consumes a_{ab} in the presence of a without modifying a.

Reactions

$$\varnothing \xrightarrow{\text{slow}} a_{\mathrm{ab}} \tag{1}$$

$$a + a_{\mathrm{ab}} \xrightarrow{\text{fast}} a_{\mathrm{ab}} \tag{2}$$

$$2\, a_{\mathrm{ab}} \xrightarrow{\text{fast}} a_{\mathrm{ab}} \tag{3}$$

Copier

Adds the value stored in y to z in the presence of g, preserving the initial quantity of y.

Reactions

$$y + g \xrightarrow{\text{slow}} y' + g \tag{4}$$

$$g + y_{\mathrm{ab}} \xrightarrow{\text{slow}} \varnothing \tag{5}$$

$$g_{\mathrm{ab}} + y' \xrightarrow{\text{slow}} y + z \tag{6}$$

Absence Indicators

$$\varnothing \xrightarrow{\text{slow}} y_{\mathrm{ab}} \tag{7}$$

$$y + y_{\mathrm{ab}} \xrightarrow{\text{fast}} y_{\mathrm{ab}} \tag{8}$$

$$2\, y_{\mathrm{ab}} \xrightarrow{\text{fast}} y_{\mathrm{ab}} \tag{9}$$

$$\varnothing \xrightarrow{\text{slow}} g_{\mathrm{ab}} \tag{10}$$

$$g + g_{\mathrm{ab}} \xrightarrow{\text{fast}} g \tag{11}$$

$$2\, g_{\mathrm{ab}} \xrightarrow{\text{fast}} g_{\mathrm{ab}} \tag{12}$$

Multiply by Two

Takes the value stored in y and doubles it in the presence of g, putting the result back in y.

Reactions

$$y + cr \xrightarrow{\text{slow}} y' + cr \tag{13}$$
$$g + y_{\text{ab}} \xrightarrow{\text{slow}} \varnothing \tag{14}$$
$$g_{\text{ab}} + y' \xrightarrow{\text{slow}} 2\,y \tag{15}$$

Absence Indicators Same as for "Copier."

Decrement

Takes the values of x and subtracts 1 from it in the presence of g, putting the result back in x.

Reactions

$$x + g \xrightarrow{\text{slow}} x' + g \tag{16}$$
$$g + x_{\text{ab}} \xrightarrow{\text{slow}} \varnothing \tag{17}$$
$$2\,x' + g'_{\text{ab}} \xrightarrow{\text{fast}} x + x' + x^{\text{rx}} \tag{18}$$
$$x^{\text{rx}} \xrightarrow{\text{slow}} \varnothing \tag{19}$$
$$x' + x^{\text{rx}}_{\text{ab}} + g'_{\text{ab}} \xrightarrow{\text{slow}} \varnothing \tag{20}$$

Absence Indicators

$$g_{\text{ab}} \xrightarrow{\text{slow}} g'_{\text{ab}} \tag{21}$$
$$g + g'_{\text{ab}} \xrightarrow{\text{fast}} g \tag{22}$$
$$2\,g'_{\text{ab}} \xrightarrow{\text{fast}} g'_{\text{ab}} \tag{23}$$
$$\varnothing \xrightarrow{\text{slow}} x_{\text{ab}} \tag{24}$$
$$x + x_{\text{ab}} \xrightarrow{\text{fast}} x \tag{25}$$
$$2\,x_{\text{ab}} \xrightarrow{\text{fast}} x_{\text{ab}} \tag{26}$$
$$\varnothing \xrightarrow{\text{slow}} x^{\text{rx}}_{\text{ab}} \tag{27}$$
$$x^{\text{rx}} + x^{\text{rx}}_{\text{ab}} \xrightarrow{\text{fast}} x^{\text{rx}} \tag{28}$$
$$2\,x^{\text{rx}}_{\text{ab}} \xrightarrow{\text{fast}} x^{\text{rx}}_{\text{ab}} \tag{29}$$
$$\varnothing \xrightarrow{\text{slow}} g_{\text{ab}} \tag{30}$$
$$g + g_{\text{ab}} \xrightarrow{\text{fast}} g \tag{31}$$
$$2\,g_{\text{ab}} \xrightarrow{\text{fast}} g_{\text{ab}} \tag{32}$$

Increment

Takes the values of x and adds 1 to it in the presence of g, putting the result back in x.

Reactions

$$x + g \xrightarrow{\text{slow}} x' + g \tag{33}$$

$$g + x_{\text{ab}} \xrightarrow{\text{slow}} \varnothing \tag{34}$$

$$2\,x' + g'_{\text{ab}} \xrightarrow{\text{fast}} x + x' + x^{\text{rx}} \tag{35}$$

$$x^{\text{rx}} \xrightarrow{\text{slow}} \varnothing \tag{36}$$

$$x' + x^{\text{rx}}_{\text{ab}} + g'_{\text{ab}} \xrightarrow{\text{slow}} 2\,x \tag{37}$$

Absence Indicators Same as for "Decrement."

Divide by Two

Takes the values of x and halves it in the presence of g, putting the result back in x.

Reactions

$$x + g \xrightarrow{\text{slow}} x' + g \tag{38}$$

$$g + x_{\text{ab}} \xrightarrow{\text{slow}} \varnothing \tag{39}$$

$$2\,x' + g'_{\text{ab}} \xrightarrow{\text{fast}} x + x^{\text{rx}} \tag{40}$$

$$x^{\text{rx}} \xrightarrow{\text{slow}} \varnothing \tag{41}$$

$$x' + x^{\text{rx}}_{\text{ab}} + g'_{\text{ab}} \xrightarrow{\text{slow}} \varnothing \tag{42}$$

Absence Indicators Same as for "Decrement."
