# Supplementary material for: Rate-Independent Constructs for Chemical Computation
Source: PLoS One. 2011 Jun 30;6(6):e21414. doi: 10.1371/journal.pone.0021414 (PMC3127954; doi:10.1371/journal.pone.0021414)
Supplement: Supporting Information S2 — Appendix: Multiplication Reactions. (PDF) [file pone.0021414.s002.pdf]

Rate-Independent Constructs for Chemical Computation

Phillip Senum, Marc Riedel
Department of Electrical and Computer Engineering, University of Minnesota, Minneapolis, Minnesota, United States
E-mail: { senu0004, mriedel }@umn.edu

Appendix: Multiplication Reactions

System Initialization We use a small set of reactions to implement the iterative loop of the operation.

$$x + x'_{\text{ab}} + y'_{\text{ab}} \xrightarrow{\text{slow}} x + g^P \tag{1}$$
$$g^P + x' \xrightarrow{\text{fast}} x' \tag{2}$$
$$g^P + y' \xrightarrow{\text{fast}} y' \tag{3}$$
$$g^P \xrightarrow{\text{slow}} g^1 + g^2 \tag{4}$$

Copying We use our copier module to implement the line of pseudo-code `z = z + y`.

$$g^1 + y \xrightarrow{\text{slow}} g^1 + y' \tag{5}$$
$$g^1 + y_{\text{ab}} \xrightarrow{\text{slow}} \varnothing \tag{6}$$
$$g^1_{\text{ab}} + y' \xrightarrow{\text{slow}} y + z \tag{7}$$

Decrement We use our decrement module to implement the line of pseudo-code `x = x - 1`.

$$x + g^2 \xrightarrow{\text{slow}} x' + g^2 \tag{8}$$
$$g^2 + x_{\text{ab}} \xrightarrow{\text{slow}} \varnothing \tag{9}$$
$$2\,x' + g'^2_{\text{ab}} \xrightarrow{\text{fast}} x' + x + x^{\text{rx}} \tag{10}$$
$$x^{\text{rx}} \xrightarrow{\text{slow}} \varnothing \tag{11}$$
$$x' + x^{\text{rx}}_{\text{ab}} + g'^2_{\text{ab}} \xrightarrow{\text{slow}} \varnothing \tag{12}$$

Absence Indicators Four absence indicators are needed by this system; they are of the same form as all others described in this paper.
