# Supplementary material for: Rate-Independent Constructs for Chemical Computation
Source: PLoS One. 2011 Jun 30;6(6):e21414. doi: 10.1371/journal.pone.0021414 (PMC3127954; doi:10.1371/journal.pone.0021414)
Supplement: Supporting Information S3 — Appendix: Raise-to-a-Power Reactions. (PDF) [file pone.0021414.s003.pdf]

Rate-Independent Constructs for Chemical Computation

Phillip Senum, Marc Riedel
Department of Electrical and Computer Engineering, University of Minnesota, Minneapolis, Minnesota, United States
E-mail: { senu0004, mriedel }@umn.edu

Appendix: Raise-to-a-Power Reactions

We present chemical reactions that implement the pseudo-code presented in the text.

System Initialization We assume that an external source injects some quantity of *begin* at the outset. This type is immediately is split into two types, g^1 and g^7, which will be used to copy x to y (for the line of code `y = x`) and to decrement p (for the line of code `p = p - 1`), respectively. This initializations takes care of the steps before the first `while` statement.

$$begin \xrightarrow{\text{fast}} g^1 + g^7 \tag{1}$$

Copy x to y $[g^1]$

$$x + g^1 \xrightarrow{\text{slow}} xy + g^1 \tag{2}$$
$$g^1 + x_{\text{ab}} \xrightarrow{\text{slow}} \varnothing \tag{3}$$
$$xy + g^1_{\text{ab}} \xrightarrow{\text{slow}} x + y \tag{4}$$

Loop Restart Our condition for restarting the main loop is that we still have p present in the system, and that we are not currently somewhere in the middle of the loop. The chemical type *done* is produced at the end of each loop from reactions 63 through 75 below. We also will wait until our post-loop cleanup in reactions 76 through 78 below is complete. At the start of each loop, we produce an injection of g^2 and g^7; these initiate the loop.

$$st_{\text{ab}} + cyc_{\text{ab}} + g^6_{\text{ab}} + stgo_{\text{ab}} + done + p \xrightarrow{\text{slow}} go^{\text{P}} + done + p \tag{5}$$
$$go^{\text{P}} + st \xrightarrow{\text{fast}} st \tag{6}$$
$$go^{\text{P}} + cyc \xrightarrow{\text{fast}} cyc \tag{7}$$
$$go^{\text{P}} + g^6 \xrightarrow{\text{fast}} g_6 \tag{8}$$
$$go^{\text{P}} + stgo \xrightarrow{\text{fast}} stgo \tag{9}$$
$$go^{\text{P}} \xrightarrow{\text{slow}} go + stgo \tag{10}$$
$$go \xrightarrow{\text{fast}} g^2 + g^7 \tag{11}$$
$$w + done \xrightarrow{\text{fast}} w \tag{12}$$
$$xw + done \xrightarrow{\text{fast}} xw \tag{13}$$
$$cyc + stgo \xrightarrow{\text{slow}} cyc \tag{14}$$

Copy x to w (once each loop) $[g^2]$ First, we take care of `w = x`.

$$x + g^2 \xrightarrow{\text{slow}} xw + g^2 \tag{15}$$
$$x_{\text{ab}} + g^2 \xrightarrow{\text{slow}} \varnothing \tag{16}$$
$$g^2_{\text{ab}} + xw \xrightarrow{\text{slow}} x + w \tag{17}$$

Loop-Running Indicator We produce a chemical type *cyc* whenever we are executing a loop. This is to ensure that our modules will not inadvertently fire when we do not wish them to do so.

$$w \xrightarrow{\text{slow}} w + cyc \tag{18}$$
$$2\,cyc \xrightarrow{\text{fast}} cyc \tag{19}$$

Multiply Loop Start The inner `while` loop is our multiply operation, handled by the next three groups of reactions.

$$w + w'_{\text{ab}} + yd_{\text{ab}} \xrightarrow{\text{slow}} w + g^{34\text{P}} \tag{20}$$
$$g^{34\text{P}} + w' \xrightarrow{\text{fast}} w' \tag{21}$$
$$g^{34\text{P}} + yd \xrightarrow{\text{fast}} yd \tag{22}$$
$$g^{34\text{P}} \xrightarrow{\text{slow}} g^3 + g^4 \tag{23}$$

Copy y to d (multiply loop) $[g^3]$

$$y + g^3 \xrightarrow{\text{fast}} yd + g^3 \tag{24}$$
$$g^3 + y_{\text{ab}} \xrightarrow{\text{slow}} \varnothing \tag{25}$$
$$g^3_{\text{ab}} + yd \xrightarrow{\text{slow}} y + d \tag{26}$$

Decrement w $[g^4]$

$$w + g^4 \xrightarrow{\text{fast}} w' + g^4 \tag{27}$$
$$g^4 + w_{\text{ab}} \xrightarrow{\text{slow}} \varnothing \tag{28}$$
$$g^4_{\text{ab}} \xrightarrow{\text{slow}} g'^4_{\text{ab}} \tag{29}$$
$$2\,w' + g'^4_{\text{ab}} \xrightarrow{\text{fast}} w' + w + w^{\text{rx}} \tag{30}$$
$$w^{\text{rx}} \xrightarrow{\text{slow}} \varnothing \tag{31}$$
$$w' + w^{\text{rx}}_{\text{ab}} + g'^4_{\text{ab}} \xrightarrow{\text{slow}} \varnothing \tag{32}$$
$$2\,g'^4_{\text{ab}} \xrightarrow{\text{slow}} g'^4_{\text{ab}} \tag{33}$$
$$g'^4_{\text{ab}} + g^4 \xrightarrow{\text{fast}} g^4 \tag{34}$$

End of Multiply Detection Once the multiplication operation has completed, we produce g^5, enabling the next step:

$$\begin{aligned}
&w_{\text{ab}} + w'_{\text{ab}} + g^2_{\text{ab}} \\
&+xw_{\text{ab}} + st_{\text{ab}} + done_{\text{ab}} \xrightarrow{\text{slow}} g^{5\text{P}} &(35)\\
&g^{5\text{P}} + w \xrightarrow{\text{fast}} w &(36)\\
&g^{5\text{P}} + w' \xrightarrow{\text{fast}} w' &(37)\\
&g^{5\text{P}} + g^2 \xrightarrow{\text{fast}} g^2 &(38)\\
&g^{5\text{P}} + xw \xrightarrow{\text{fast}} xw &(39)\\
&g^{5\text{P}} + st \xrightarrow{\text{fast}} st &(40)\\
&g^{5\text{P}} + done \xrightarrow{\text{fast}} done &(41)\\
&g^{5\text{P}} + cyc \xrightarrow{\text{slow}} g^5 + cyc &(42)
\end{aligned}$$

Clear y $[g^5]$ We must take care of the lines `y = d` and `d = 0`. First, we clear our previous quantity of y.

$$\begin{aligned}
&g^5 + y \xrightarrow{\text{slow}} g^5 &(43)\\
&y_{\text{ab}} + g^5 \xrightarrow{\text{slow}} \varnothing &(44)
\end{aligned}$$

Inhibit production of g^5 We stop production of g^5 so that we may preserve the quantity of y that we are going to receive from d.

$$\begin{aligned}
&y_{\text{ab}} + yd_{\text{ab}} \xrightarrow{\text{slow}} st^{\text{P}} &(45)\\
&st^{\text{P}} + y \xrightarrow{\text{fast}} y &(46)\\
&st^{\text{P}} + yd \xrightarrow{\text{fast}} yd &(47)\\
&st^{\text{P}} + g^5 \xrightarrow{\text{slow}} st + g^5 &(48)
\end{aligned}$$

Set y **to** d $[g^6]$ Finally, we transfer d to y, clearing d in the process.

$$\begin{aligned}
&y_{\text{ab}} + g^5_{\text{ab}} + yd_{\text{ab}} \xrightarrow{\text{slow}} g^{6\text{P}} &(49)\\
&g^{6\text{P}} + y \xrightarrow{\text{fast}} y &(50)\\
&g^{6\text{P}} + g^5 \xrightarrow{\text{fast}} g^5 &(51)\\
&g^{6\text{P}} + yd \xrightarrow{\text{fast}} yd &(52)\\
&g^{6\text{P}} + d + st \xrightarrow{\text{slow}} g^6 + d + st &(53)\\
&g^6 + d \xrightarrow{\text{slow}} g^6 + y &(54)
\end{aligned}$$

Decrement $p\ \left[g^7\right]$ The decrement of p is used several in two distinct cases, but we only need one instance of the module for our system.

$$p + g^7 \xrightarrow{\text{fast}} p' + g^7 \tag{55}$$
$$g^7 + p_{\text{ab}} \xrightarrow{\text{slow}} \varnothing \tag{56}$$
$$g^7_{\text{ab}} \xrightarrow{\text{slow}} g'^7_{\text{ab}} \tag{57}$$
$$2\,p' + g'^7_{\text{ab}} \xrightarrow{\text{fast}} p' + p + p^{\text{rx}} \tag{58}$$
$$p^{\text{rx}} \xrightarrow{\text{slow}} \varnothing \tag{59}$$
$$p' + p^{\text{rx}}_{\text{ab}} + g'^7_{\text{ab}} \xrightarrow{\text{slow}} \varnothing \tag{60}$$
$$2\,g'^7_{\text{ab}} \xrightarrow{\text{slow}} g'^7_{\text{ab}} \tag{61}$$
$$g'^7_{\text{ab}} + g^7 \xrightarrow{\text{fast}} g^7 \tag{62}$$

End-of-Loop Detection We know that we have finished a loop when all operations within and prior to the loop have completed.

$$\begin{aligned} &yd_{\text{ab}} + d_{\text{ab}} + go_{\text{ab}} + g^2_{\text{ab}} \\ &+xw_{\text{ab}} + begin_{\text{ab}} + g^1_{\text{ab}} \\ &+xy_{\text{ab}} + g^7_{\text{ab}} + p'_{\text{ab}} \xrightarrow{\text{slow}} done^{\text{P}} \end{aligned} \tag{63}$$
$$done^{\text{P}} + yd \xrightarrow{\text{fast}} yd \tag{64}$$
$$done^{\text{P}} + d \xrightarrow{\text{fast}} d \tag{65}$$
$$done^{\text{P}} + go \xrightarrow{\text{fast}} go \tag{66}$$
$$done^{\text{P}} + g^2 \xrightarrow{\text{fast}} g^2 \tag{67}$$
$$done^{\text{P}} + xw \xrightarrow{\text{fast}} xw \tag{68}$$
$$done^{\text{P}} + begin \xrightarrow{\text{fast}} begin \tag{69}$$
$$done^{\text{P}} + g^1 \xrightarrow{\text{fast}} g^1 \tag{70}$$
$$done^{\text{P}} + xy \xrightarrow{\text{fast}} xy \tag{71}$$
$$done^{\text{P}} + g^7 \xrightarrow{\text{fast}} g^7 \tag{72}$$
$$done^{\text{P}} + p' \xrightarrow{\text{fast}} p' \tag{73}$$
$$done^{\text{P}} \xrightarrow{\text{slow}} done \tag{74}$$
$$2\,done \xrightarrow{\text{fast}} done \tag{75}$$

Post-Loop Cleanup Finally, we reset the system back to its initial state.

$$st + done \xrightarrow{\text{fast}} done \tag{76}$$
$$g^6 + done \xrightarrow{\text{fast}} done \tag{77}$$
$$cyc + done \xrightarrow{\text{fast}} done \tag{78}$$

Absence Indicators

Twenty-five absence indicators are used by the reactions above. They are generated by the method outlined in the paper and omitted here to save space.
