# Supplementary material for: Rate-Independent Constructs for Chemical Computation
Source: PLoS One. 2011 Jun 30;6(6):e21414. doi: 10.1371/journal.pone.0021414 (PMC3127954; doi:10.1371/journal.pone.0021414)
Supplement: Supporting Information S4 — Appendix: Exponentiation Reactions. (PDF) [file pone.0021414.s004.pdf]

Rate-Independent Constructs for Chemical Computation

Phillip Senum, Marc Riedel
Department of Electrical and Computer Engineering, University of Minnesota, Minneapolis, Minnesota, United States
E-mail: { senu0004, mriedel }@umn.edu

Appendix: Exponentiation Reactions

We present chemical reactions that implement the pseudo-code presented in the text.

System Initialization As with our multiplication module, we have a small set of reactions to control the overall timing.

$$x + x'_{\mathrm{ab}} + y'_{\mathrm{ab}} \xrightarrow{\text{slow}} x + g^P \tag{1}$$
$$g^P + x' \xrightarrow{\text{fast}} x' \tag{2}$$
$$g^P + y' \xrightarrow{\text{fast}} y' \tag{3}$$
$$g^P \xrightarrow{\text{slow}} g^1 + g^2 \tag{4}$$

Doubling We use a slight variation of our copier module to implement the line of pseudo-code `y = 2 * y`.

$$g^1 + y \xrightarrow{\text{slow}} g^1 + y' \tag{5}$$
$$g^1 + y_{\mathrm{ab}} \xrightarrow{\text{slow}} \varnothing \tag{6}$$
$$g^1_{\mathrm{ab}} + y' \xrightarrow{\text{slow}} 2\,y \tag{7}$$

Decrement As with our multiplication module, we decrement `x` once each loop.

$$x + g^2 \xrightarrow{\text{slow}} x' + g^2 \tag{8}$$
$$g^2 + x_{\mathrm{ab}} \xrightarrow{\text{slow}} \varnothing \tag{9}$$
$$2\,x' + g'^2_{\mathrm{ab}} \xrightarrow{\text{fast}} x' + x + x^{\mathrm{rx}} \tag{10}$$
$$x^{\mathrm{rx}} \xrightarrow{\text{slow}} \varnothing \tag{11}$$
$$x' + x^{\mathrm{rx}}_{\mathrm{ab}} + g'^2_{\mathrm{ab}} \xrightarrow{\text{slow}} \varnothing \tag{12}$$

Absence Indicators Four absence indicators are needed by this system; they are of the same form as all others described in this paper.
