# Supplementary material for: Rate-Independent Constructs for Chemical Computation
Source: PLoS One. 2011 Jun 30;6(6):e21414. doi: 10.1371/journal.pone.0021414 (PMC3127954; doi:10.1371/journal.pone.0021414)
Supplement: Supporting Information S5 — Appendix: Logarithm Reactions. (PDF) [file pone.0021414.s005.pdf]

Rate-Independent Constructs for Chemical Computation

Phillip Senum, Marc Riedel
Department of Electrical and Computer Engineering, University of Minnesota, Minneapolis, Minnesota, United States
E-mail: { senu0004, mriedel }@umn.edu

Appendix: Logarithm Reactions

We present chemical reactions that implement the pseudo-code presented in the text.

System Initialization As with our other modules, we have a small set of reactions to control the overall timing of our system.

$$2\,x + x'_{\mathrm{ab}} + y'_{\mathrm{ab}} \xrightarrow{\mathrm{slow}} 2\,x + g^P \quad (1)$$
$$g^P + x' \xrightarrow{\mathrm{fast}} x' \quad (2)$$
$$g^P + y' \xrightarrow{\mathrm{fast}} y' \quad (3)$$
$$g^P \xrightarrow{\mathrm{slow}} g^1 + g^2 \quad (4)$$

Halving We use a slight variation of our decrement module to implement the operation `x = x / 2`.

$$x + g^1 \xrightarrow{\mathrm{slow}} x' + g^1 \quad (5)$$
$$g^1 + x_{\mathrm{ab}} \xrightarrow{\mathrm{slow}} \varnothing \quad (6)$$
$$2\,x' + g'^1_{\mathrm{ab}} \xrightarrow{\mathrm{fast}} x + x^{\mathrm{rx}} \quad (7)$$
$$x^{\mathrm{rx}} \xrightarrow{\mathrm{slow}} \varnothing \quad (8)$$
$$x' + x^{\mathrm{rx}}_{\mathrm{ab}} + g'^1_{\mathrm{ab}} \xrightarrow{\mathrm{slow}} \varnothing \quad (9)$$
$$(10)$$

Increment We use our increment module to implement the line `y = y + 1`. We set y to be 1 initially.

$$y + g^2 \xrightarrow{\mathrm{slow}} y' + g^2 \quad (11)$$
$$g^2 + y_{\mathrm{ab}} \xrightarrow{\mathrm{slow}} \varnothing \quad (12)$$
$$2\,y' + g'^2_{\mathrm{ab}} \xrightarrow{\mathrm{fast}} y' + y + y^{\mathrm{rx}} \quad (13)$$
$$y^{\mathrm{rx}} \xrightarrow{\mathrm{slow}} \varnothing \quad (14)$$
$$y' + y^{\mathrm{rx}}_{\mathrm{ab}} + g'^2_{\mathrm{ab}} \xrightarrow{\mathrm{slow}} 2\,y \quad (15)$$
$$(16)$$

Cleanup Once the module has completed, we decrement y by one, storing the result in y_f.

$$x'_{\text{ab}} + y'_{\text{ab}} \xrightarrow{\text{slow}} done^{\text{P}} \tag{17}$$

$$done^{\text{P}} + 2\,x \xrightarrow{\text{fast}} 2\,x \tag{18}$$

$$done^{\text{P}} + x' \xrightarrow{\text{fast}} x' \tag{19}$$

$$done^{\text{P}} + y' \xrightarrow{\text{fast}} y' \tag{20}$$

$$done^{\text{P}} \xrightarrow{\text{slow}} done \tag{21}$$

$$2\,done \xrightarrow{\text{fast}} done \tag{22}$$

$$done + 2\,y \xrightarrow{\text{slow}} y + y_f \tag{23}$$

Absence Indicators Two special absence indicators are used by the halving and increment modules above; a total of 13 are needed for the system to function properly. They are of the same form as all other absence indicators, described in the paper.
